# Chronic Idiopathic Intestinal Pseudo-Obstruction

**DOI:** 10.7759/cureus.16563

**Published:** 2021-07-22

**Authors:** Xuanzhen Piao, Grace W Ying, Michael J Chaney, Shirly Samuel, Artem Sharko, Farah Zahra

**Affiliations:** 1 Medicine, Chicago Medical School Rosalind Franklin University of Medicine and Science, North Chicago, USA; 2 Internal Medicine, Chicago Medical School Internal Medicine Residency Program at Northwestern McHenry Hospital, McHenry, USA

**Keywords:** bowel obstruction, pseudo-obstruction, gastrojejunostomy, gastric outlet obstruction, delayed gastric emptying, biliary ductal dilatation

## Abstract

Chronic intestinal pseudo-obstruction (CIPO) is a rare, potentially debilitating gastrointestinal (GI) condition characterized by symptoms of intestinal obstruction with the absence of anatomic lesions. In this report, we present a case of an 86-year-old female who presented with severe abdominal discomfort, nausea, and vomiting for two weeks prior to presentation. Imaging studies revealed severe gastric distension with a lack of anatomic lesions. The patient was ultimately diagnosed with chronic idiopathic intestinal pseudo-obstruction (CIIP). The purpose of this case report is to raise awareness of this condition in the medical literature and discuss the epidemiology, pathophysiology, clinical manifestations, diagnostic workup, and treatment options of this disorder.

## Introduction

Chronic intestinal pseudo-obstruction (CIPO) is defined as mechanical bowel obstruction in the absence of an organic lesion. CIPO has an incidence of 0.21 and 0.24 per 100,000 men and women, respectively; it is typically diagnosed in patients over the age of 60 years [1]. It can present with various clinical pictures depending on the site in which there is loss of motility from muscle or nerve abnormalities, but it is commonly manifested as noncolicky abdominal pain and distension aggravated by food intake. CIPO is generally classified into primary and secondary CIPO [2]. Primary CIPO is further subclassified into myopathic and neuropathic CIPO. These are usually seen in congenital or familial cases, where there are intrinsic muscle or nerve abnormalities within the gastrointestinal (GI) tract resulting in weakened or unsynchronized peristalsis [2]. Secondary CIPO is usually caused by other underlying systemic, metabolic, or organic conditions such as autoimmune disorders, endocrine disorders, neurological disorders, malignancies, and certain infections [2]. Worth noting, medications such as tricyclic antidepressants, anticholinergic agents, or narcotics can also result in secondary CIPO. Lastly, chronic idiopathic intestinal pseudo-obstruction (CIIP) is referred to patients with an unknown cause of CIPO, as seen in our patient. While CIPO is not considered an acutely dangerous disease, the symptoms and difficulty in achieving adequate nutritional status can be debilitating for patients. In this case, we report an elderly female who presented to the emergency department with two weeks of abdominal pain, nausea, and vomiting.

## Case presentation

The patient is an 86-year-old woman without significant past medical history who presented to the emergency department with a complaint of severe epigastric discomfort accompanied by intermittent nausea and vomiting for two weeks before presentation. She stated that she was having persistent painless abdominal bloating. She reported having worsening nausea and vomiting with oral intake to the point that she could no longer keep any solid or liquid down without vomiting. The associated symptoms included heartburn while taking omeprazole and dyspepsia for approximately two years. She further denied fever, chills, night sweats, unintentional weight loss, chest pain, shortness of breath, odynophagia, hematemesis, constipation, diarrhea, melena, or hematochezia. She denied personal history of diabetes, myopathy, neuropathy, or malignancy. Her past surgical history was significant for hysterectomy. She was a former smoker and denied drinking alcohol or using recreational drugs. Her family history was significant for colon cancer in her mother. Her last colonoscopy was performed about thirty-five years ago, which was unremarkable. She never had an esophagogastroduodenoscopy (EGD) done in the past. 

On presentation, she had a temperature of 97.5°F, heart rate of 80 beats per minute, respiratory rate of 18 breaths per minute, blood pressure of 99/56 mmHg, and oxygen saturation of 99% on room air. On physical exam, she had dry oral mucous membrane. The abdomen was nondistended and nontender with the presence of bowel sound. The initial lab work, including complete blood count and comprehensive metabolic panel, was remarkable for creatinine of 1.48 with a baseline of 0.85-0.95 and blood urea nitrogen of 28. Other relevant laboratory data, including those collected later at a tertiary hospital, were all within normal limits. These include A1c, lipase, TSH, CEA, CA 19-9, immunoglobulin G subclass 4, antinuclear antibody, anti-double-stranded deoxyribonucleic acid antibody, anti-centromere antibody, anti-Ro antibody, and anti-La antibody.

**Figure 1:**
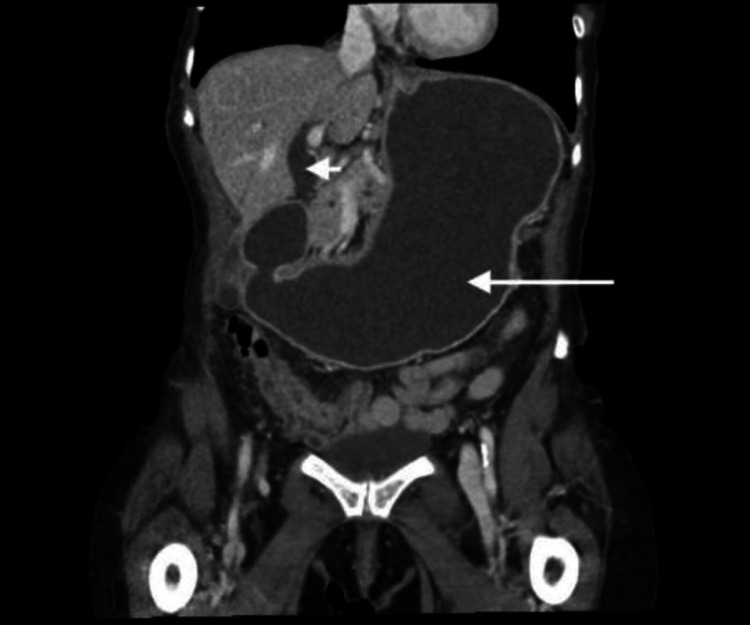
CT abdomen and pelvis with contrast revealing moderate to severely distended stomach (white arrow) with abrupt caliber change in the proximal duodenum. There is also moderate dilatation of the common bile duct (arrowhead) with abrupt caliber change at the ampulla, likely related to the duodenal findings. CT: computed tomography.

**Figure 2:**
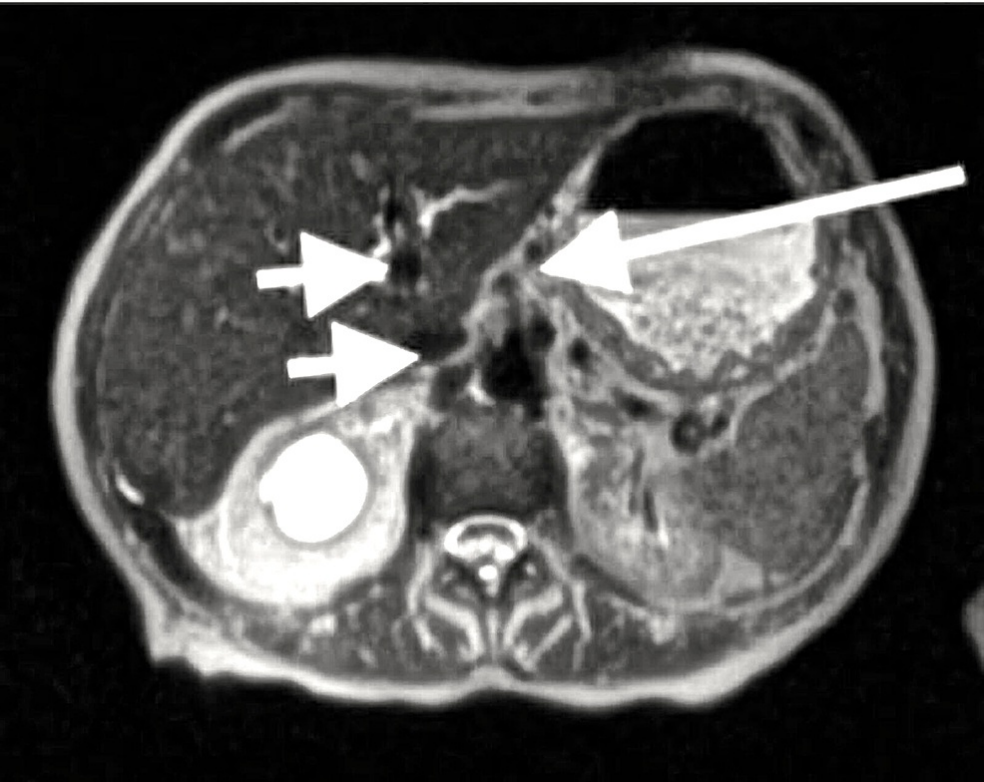
MRI and MRCP abdomen without contrast showing distended stomach to the level of the mid descending segment of the duodenum, at which level there is diffuse mucosal wall thickening (white arrow). There is also intra and extrahepatic biliary ductal dilatation and prominence of the common bile duct to the level of the ampulla (arrowhead). No extraluminal mass is identified. MRI: magnetic resonance imaging; MRCP: magnetic resonance cholangiopancreatography.

The abdominal and pelvic computed tomography (CT) with contrast revealed moderate to severely distended stomach and dilatation of the common bile duct with caliber change at the ampulla (Figure 1). The subsequent EGD was negative for esophageal blockage but positive for gastric outlet obstruction in the second portion of the duodenum. The stomach biopsy revealed reactive gastropathy and negative findings for Helicobacter pylori organisms. The duodenal biopsy demonstrated nonspecific chronic inflammation involving lamina propria with immunohistochemical stain for CD3 negative for increased intraepithelial T lymphocytes, making celiac disease unlikely. Follow-up abdominal magnetic resonance imaging (MRI) and magnetic resonance cholangiopancreatography (MRCP) without contrast did not reveal any extraluminal mass (Figure 2). She also underwent a fluoroscopy upper gastrointestinal tract radiography with air contrast, which showed delayed gastric emptying without evidence of high-grade stricture or gastric mucosal abnormality.

The patient was initially started on suction via a nasogastric tube to help with symptom control. Given the acuity and severity of the situation, as she could no longer keep any solid or liquid down without vomiting, conservative management was not started. Instead, she underwent exploratory laparotomy, mobilization of the duodenum with Kocher maneuver, and gastrojejunostomy with surgical biopsies negative for primary duodenal or ampullary malignancy. Post-operative abdominal CT with contrast showed focal dilation of small bowel in the left abdomen without definite evidence of obstruction. With multiple imaging findings supporting fibrotic area narrowing in the distal second portion of the duodenum and essentially extensive negative laboratory workup, the diagnosis of CIIP was made. The post-operative course was uncomplicated. By postoperative day six, she had good pain control with oral medications, tolerated a low-fiber diet, had a complete return of bowel function, and was subsequently discharged home on tramadol as needed. 

## Discussion

Clinical manifestation of CIPO mainly depends on its location and extension within the GI tract. Generally, patients present with noncolicky abdominal pain and distension that is aggravated by eating [3]. Other associated symptoms may include nausea, vomiting, constipation, and diarrhea. The diagnosis of CIPO is challenging as the clinical symptoms are non-specific and often overlap with other conditions such as gastroparesis, functional constipation, drug toxicity, and hypothyroidism [3]. For example, in gastroparesis, there is delayed gastric emptying due to partial paralysis of the stomach from nerve injury without obstruction in the stomach or intestines. In other words, supportive treatment with agents such as metoclopramide, erythromycin or antiemetics is usually sufficient to keep the condition under control. In contrast, the presence of intestinal obstruction with dilatation and air-fluid level without anatomic lesion on radiographic imaging is essential to diagnose CIPO [2]. An EGD or colonoscopy can be performed to rule out any intraluminal or extraluminal cause of obstruction [4]. Following endoscopic studies, scintigraphy or manometry studies can be used to supplement the diagnosis of CIPO by assessing the rate at which materials travel through the GI tract or the contractions within the intestinal tract, respectively [2]. Lastly, histology studies of the GI tract can be performed to rule out other underlying conditions such as infections [5]. 

The management of CIPO requires coordinated efforts from a multidisciplinary team [6]. The main goals of treatment are to provide pain relief, increase GI motility, maintain good nutritional status, and prevent complications of motility loss such as bacterial overgrowth [7]. Medications can be used to control specific symptoms in individuals with CIPO [2]. Visceral or abdominal pain is the primary concern in most patients, which can be addressed by low-dose gabapentin [8]. Opioids are usually not recommended as they inhibit GI peristalsis, which may exacerbate the condition [3]. Prokinetics such as erythromycin and octreotide have been prescribed to improve GI motility by increasing the frequency of contractions in the small intestines [9]. Enteral or total parenteral nutrition (TPN) can be initiated to maintain basic nutritional requirements for patients who cannot eat due to the severity of their disorder [2]. Considering the cost and associated complications of TPN, the enteral pathway is always attempted first. Antibiotics such as amoxicillin-clavulanic acid, ciprofloxacin, and doxycycline can be prescribed to treat bacterial infections and alleviate diarrhea and bloating [3]. Some individuals with CIPO can benefit from intestinal decompression as the procedure can reduce the pressure within the GI tract leading to relief of abdominal pain and swelling. In patients with severe symptoms of CIPO, surgical removal of a segment of the intestine may be necessary to completely obliterate symptoms such as retching, vomiting, and abdominal distention. Intestinal transplantation is the definitive therapy for patients with CIPO who do not respond to other treatments [2]. In individuals with secondary CIPO, management of the underlying disorder should be attempted. Lastly, dietary modifications such as a low-fat, low-fiber diet and small, frequent meals are encouraged in patients with CIPO.

## Conclusions

CIPO is a rare syndrome in which symptoms of GI obstruction occur in the absence of an organic lesion. Because of its rarity and the fact that the symptoms can at times be non-specific or without an apparent cause, the diagnosis can be easily missed. Therefore, CIPO and CIIP should be part of the clinician's diagnostic differentials in patients presenting with these varied and less specific GI signs and symptoms.
